# Distractor onset but not preparation time affects the frequency of task confusions in task switching

**DOI:** 10.3389/fpsyg.2015.01671

**Published:** 2015-10-27

**Authors:** Marco Steinhauser, Miriam Gade

**Affiliations:** Department of Psychology, Catholic University of Eichstätt-Ingolstadt, Eichstätt, Germany

**Keywords:** errors, task switching, task preparation, cognitive control, error detection

## Abstract

When participants rapidly switch between tasks that share the same stimuli and responses, task confusions (i.e., the accidental application of the wrong task) can occur. The present study investigated whether these task confusions result from failures of endogenous control (i.e., from ineffective task preparation) or from failures of exogenous control (i.e., from stimulus-induced task conflicts). The frequency of task confusions was estimated by considering the relative proportion of distractor errors, that is, errors that result when participants erroneously respond to the distractor associated with the alternative task. In Experiment 1, the efficiency of exogenous control was manipulated by varying the temporal order of target and distractor presentation. In Experiment 2, the efficiency of endogenous control was manipulated by varying the time available for preparing the task in advance. It turned out that only the efficiency of exogenous control but not the efficiency of endogenous control influenced the proportion of distractor errors. Accordingly, task confusions are more related to failures in exogenous control.

## Introduction

Current theories on cognitive control assume that goal-directed behavior is achieved by controlling the task set. While task set refers to a configuration of the cognitive system that allows for executing a certain task, task-set control denotes the process by which task sets are selected and implemented. Task-set control can occur endogenously, when control processes reconfigure the cognitive system according to current goals and intentions (Rogers and Monsell, 1995; Meiran, 1996), but also exogenously, when external stimuli activate a task set according to acquired stimulus-task associations (e.g., Steinhauser and Hübner, 2007, 2009; Yamaguchi and Proctor, 2011). In the present study, we investigated how failures in task-set control can lead to task confusions, i.e., to the accidental execution of the wrong task. More specifically, we asked whether task confusions are due to failures of endogenous control, exogenous control, or both. An answer to this question can provide valuable information about control processes involved in task-set control.

Task-set control is typically investigated using the task-switching paradigm (Kiesel et al., 2010), which requires multiple tasks to be performed in random or pre-specified order. When performance on task-switch trials is compared with that on task-repetition trials, then so-called *switch costs* are observed, which are assumed to reflect task-set reconfiguration (Rogers and Monsell, 1995; Meiran, 1996; Oberauer et al., 2013) but also memory processes like priming or associative strengthening of tasks (Allport et al., 1994; Meiran, 2000; Schuch and Koch, 2003; Steinhauser and Hübner, 2006; Philipp et al., 2007). Evidence for the occurrence of task confusions came from studies showing that performance after errors is better for task switches than for task repetitions (Steinhauser and Hübner, 2006, 2008; Steinhauser, 2010; Desmet et al., 2012). This can be explained by assuming that some of these errors are due to task confusions, which turn a subsequent task switch into a task repetition, and vice versa. Moreover, a recent study revealed dissociable neural correlates of task confusions and response confusions (Desmet et al., 2011).

The question arises how these task confusions emerge. Basically, there are two possible sources which are related to the two types of control mentioned above: First, a task confusion can occur because endogenous control has failed, for instance, because the wrong task set is retrieved during preparation (Altmann and Gray, 2008; Oberauer et al., 2013) or the control system fails to engage in endogenous reconfiguration (De Jong, 2000). Second, a task confusion can occur because exogenous control has failed, that is, because the stimulus activated the wrong task set, for instance, because irrelevant stimulus features associated with the wrong task capture attention (Steinhauser and Hübner, 2007, 2009) and therefore trigger instantaneous task-set retrieval from memory (Yamaguchi and Proctor, 2011).

To determine the source of task confusions, it would be necessary to directly measure the frequency of task confusions. One way to achieve this is to use non-overlapping response sets. In a recent study, Meiran and Daichman (2005) assigned a different hand to each task, and measured the frequency of task confusions by considering the number of hand confusions. Their results suggested that task confusions are influenced by preparation time, which implies that they are due to failures of endogenous control. However, it is not clear whether hand confusions are really due to task confusions, or whether they simply reflect failures in motor preparation. In a further step, Meiran and Daichman (2005) confirmed their results for tasks with overlapping responses using multinomial modeling. Their modeling results showed that a short preparation time led to a large frequency of task confusion on task switches while this was considerably reduced with a long preparation time. However, conclusions from multinomial models depend strongly on implicit theoretical assumptions, like, e.g., the statistical independence of latent events (e.g., Chen et al., 2015).

To overcome these problems, the present study made use of a novel method. We developed a three-choice paradigm in which task confusions and response confusions were differentially associated with different error types. Whereas one task consisted of classifying a symbol as number, letter, or non-alphanumeric character (e.g., %), another task consisted of classifying a picture as fruit, vehicle, or animal. Each response key was assigned to one category for each task (e.g., the left key was pressed for animal and letter). The stimuli consisted of a target associated with the relevant task and a distractor associated with the irrelevant task, but only stimuli were used for which target and distractor were associated with different responses. This allowed for distinguishing between two types of errors (see also Maier et al., 2012; Maier and Steinhauser, 2013): Participants could erroneously press the key associated with the distractor (*distractor error*) or the key associated with neither the target nor the distractor (*non-distractor error*).

Crucially, the relative proportion of distractor errors (among all errors) can serve as a correlate of the frequency of task confusions. If only the response is confused (while the correct task set is applied), this should equally often lead to a distractor error and a non-distractor error. If, however, the task is confused (and the correct response for that task is produced), this should always lead to a distractor error. This implies that the relative proportion of distractor errors is positively correlated with the relative proportion of task confusions. As a consequence, although the frequency of task confusions cannot be exactly estimated in this way, the proportion of distractor errors should be a meaningful correlate of this frequency, and thus, can be used to measure the susceptibility to task confusions in a condition.

In two experiments, this rationale was used to investigate whether task confusions are related to failures of endogenous task-set control or to failures of exogenous task-set control. To this end, it was examined whether manipulations of the efficiency of each control type affects the susceptibility to task confusions. In Experiment 1, the efficiency of exogenous control was manipulated by varying the temporal order in which the target and the distractor appeared. In Experiment 2, the efficiency of endogenous control was manipulated by varying the time for preparing the task in advance. If task confusions were due to failures of exogenous task-set control, the relative proportion of distractor errors should depend on the efficiency of exogenous task-set control. Similarly, if task confusions were due to failures of endogenous control, the relative proportion of distractor errors should depend on the efficiency of endogenous control. Of course, both predictions could hold because both assumptions are not mutually exclusive.

In addition, we were interested in the detectability of errors that result from task confusions. In one experiment, Steinhauser and Hübner (2006) required participants to signal their errors by pressing a neutral signaling key whenever they detected an error. It turned out that even detected errors lead to switch benefits on the subsequent trial. Although this implies that errors due to task confusions are principally detectable, one could hypothesize that error detection is harder for these errors than for errors due to response confusions. Current theories on error detection assume that errors are detected because the correct response becomes activated after an error has occurred (Yeung et al., 2004; Steinhauser et al., 2008). This should be more difficult when the error is due to task confusion, because activating the correct response requires that first the correct task is activated. Accordingly, we hypothesize that distractor errors should be less detectable than non-distractor errors.

## Experiment 1

Studies on selective attention have shown that presenting a target feature prior to a distractor feature increases the probability that the distractor feature activates a response (Glaser and Glaser, 1982). The same should hold for the task. The relevant task set should be activated more strongly by the stimulus when the target appeared first, whereas the irrelevant task set should be activated more strongly when the distractor appeared first. Accordingly, target-distractor order can be used to manipulate the efficiency of exogenous task-set control. If the efficiency of exogenous control influences the susceptibility to task confusions, the proportion of distractor errors should be increased when the distractor appears first.

### Materials and Methods

#### Participants

Twenty-two participants (15 female) between 19 and 28 years of age (mean 21.9) with normal or corrected-to-normal vision participated in the experiment. Participants were recruited at the University of Konstanz and were paid 7 Euro per hour. The study was conducted in accordance with the Declaration of Helsinki and the guidelines of the ethics committee at the University of Konstanz, and informed consent was acquired from all participants.

#### Apparatus

The stimuli were presented on a 21-inch color monitor. An IBM-compatible PC controlled stimulus presentation and response registration.

#### Stimuli

Each stimulus consisted of a character and a picture presented horizontally arranged. The set of characters consisted of four letters (A, B, C, D), four numerals (1, 2, 3, 4), and four symbols ($, %, &, ?), taken from Arial font. The set of pictures consisted of four animals (bird, cat, dog, mouse), four fruits (apple, banana, cherry, pear), and four vehicles (aircraft, bike, car, sailboat), taken from the Snodgrass-Vanderwart Set of Standardized Pictures (Snodgrass and Vanderwart, 1980). Only characters and pictures were combined that were associated with different responses (i.e., incongruent stimuli). These combinations were realized in each possible order (left/right). Moreover, because the two stimulus parts faded in asynchronously on the screen, each stimulus was realized with the picture appearing first or with the character appearing first. Altogether, this resulted in 384 stimuli. A circle and a square were used as cues. Cues and stimuli were presented in white color on a black background.

#### Design and Procedure

On each trial, participants had to apply one of two judgments. For the character judgment, the character in the stimulus had to be classified as “letter,” “numeral,” or “symbol.” For the picture judgment, the picture in the stimulus had to be classified as “animal,” “fruit,” or “vehicle.” Responses were given with the right hand by pressing the “arrow left” key with the index finger for the categories “letter” and “animal,” the “arrow down” key with the middle finger for the categories “numeral” and “fruit,” or the “arrow right” key with the ring finger for the categories “symbol” and “vehicle.” In addition, participants were required to signal their errors. They were instructed to press the space bar with their left hand whenever they think that their first response was an error.

Each trial started with the presentation of the cue for 300 ms followed by a blank screen for 900 ms. Then, the first stimulus part (target or distractor stimulus) appeared. After a delay of 100 ms, the second stimulus part appeared. The full stimulus remained on the screen for 150 ms followed by a blank screen. 1000 ms after the response, a new trial started. If a signaling response or a correction response occurred during this interval, a new interval of 1000 ms was started. No feedback on the accuracy of the response was provided.

Participants worked through six test blocks of 144 trials, resulting in a total amount of 864 trials. Within each block, the sequence of tasks (character vs. picture) was randomized. Four practice blocks of 72 trials preceded the test blocks. In the first practice block, the task rules were learnt without time pressure. In the second practice block, participants were instructed to respond as fast as possible. At the end of this and the following blocks, participants were encouraged to respond more quickly when the error rate dropped below 15%. In the third practice block, error signaling was introduced.

### Results

Trials were classified according to whether the response corresponded to the target (correct), the distractor (distractor errors) or none of the stimulus elements (non-distractor error). For the analysis of response times (RT), trials were excluded with RTs deviating more than three standard deviations from the mean computed for each condition and participant.

#### RTs and Error Rates

**FIGURE 1:**
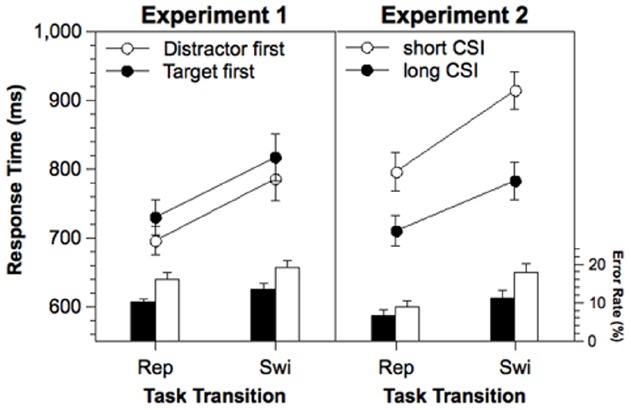
**RTs and error rates in Experiments 1 and 2 as a function of Task Transition, Stimulus Order (Experiment 1), and Cue-Stimulus Interval (Experiment 2).** Error bars represent standard errors of the mean. Rep, repetition; Swi, Switch; ms, milliseconds.

Response times of correct responses and error rates were analyzed in a two-way ANOVA with repeated measurement on the variables Task Transition (task switch, task repetition) and Stimulus Order (target first, distractor first). The results are presented in Figure 1, left panel. In the RTs, a significant main effect of Task Transition was obtained, indicating significant switch costs (88 ms), *F*(1,21) = 26.9, *p* < 0.001. The effect of Stimulus Order was only marginally significant, *F*(1,21) = 4.18, *p* < 0.06. RTs were shorter when the distractor appeared first (741 ms) than when the target appeared first (774 ms). In the error rates, Task Transition as well as Stimulus Order reached significance. There were significant error switch costs (3.3%), *F*(1,21) = 16.5, *p* < 0.001, and the error rate was larger when the distractor appeared first (17.5%) as compared to when the target appeared first (11.8%), *F*(1,21) = 7.28, *p* < 0.05.

#### Proportion Distractor Errors

**FIGURE 2:**
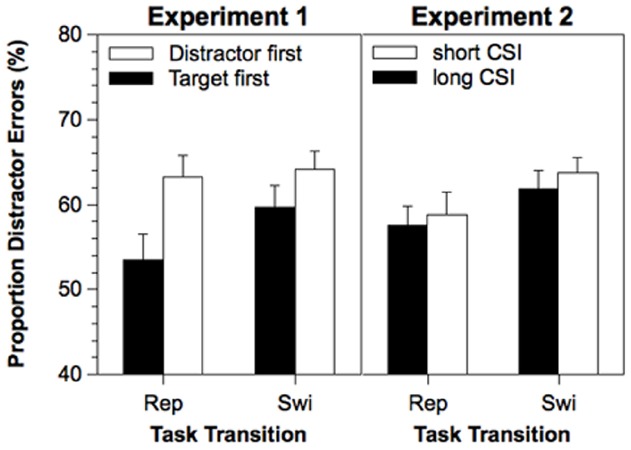
**Proportion of distractor errors in Experiment 1 and 2 as a function of Task Transition, Stimulus Order (Experiment 1), and Cue-Stimulus Interval (Experiment 2).** Error bars represent standard errors of the mean. Rep, repetition; Swi, Switch; ms, milliseconds.

The relative proportion of distractor errors (among all errors) was analyzed in a similar analysis (see, Figure 2, left panel). The mean proportion of distractor errors was 60.1%, which was significantly above 50%, *t*(21) = 6.78, *p* < 0.001. The main effect of Stimulus Order reached significance, *F*(1,21) = 5.67, *p* < 0.05. The proportion of distractor errors was increased when the distractor appeared first (63.7%) as compared to when the target appeared first (56.5%). The main effect of Task Transition was only marginally significant, *F*(1,21) = 3.56, *p* < 0.07. The proportion of distractor errors was slightly increased on task switches (61.9%) as compared to task repetitions (58.3%). The interaction between Task Transition and Stimulus Order was not significant (*F*<1).

#### Error Signaling

One participant had to be excluded from the analysis of signaling latencies due to a low number of signaled errors. The relative frequency of signaled errors (among all errors) as well as the signaling latency (i.e., the time between the first response and the signaling response) was compared between distractor errors and non-distractor errors. Distractor errors were signaled less frequently (63.3%) than non-distractor errors (75.5%), *F*(1,21) = 23.2, *p* < 0.001. Moreover, signaling latencies were significantly longer for distractor errors (618 ms) than for non-distractor errors (546 ms), *F*(1,20) = 8.53, *p* < 0.05.

### Discussion

The fact that the proportion of distractor errors exceeded 50% shows that a substantial number of distractor errors were due to task confusions. If only response confusions had occurred, then each of the two wrong alternative responses should be equally frequent, leading to a proportion of 50% for each error type. Most important, however, the data indicate that stimulus order influenced the proportion of distractor errors, and thus, the susceptibility to task confusions. When the distractor appeared first, not only the error rate was increased but also the proportion of distractor errors. These results clearly support the view that the susceptibility to task confusion is affected by the efficiency of exogenous control, that is, by the efficiency with which target and distractor can activate their associated tasks (see Yamaguchi and Proctor, 2011; Schneider, 2015; for recent discussion of such rapid task-set activation). A somewhat unexpected result is the trend toward shorter RTs when the distractor appeared first. This could reflect that the early distractor occasionally elicits a fast guess that is sometimes correct. This further shows that target-distractor order is a viable method to manipulate exogenous task-set control. Finally, we were interested in the detectability of the two error types. As predicted, non-distractor errors were detected more frequently and more quickly than distractor errors.

## Experiment 2

Experiment 2 investigated whether task confusions also result from failures of endogenous control. Endogenous control was manipulated by varying preparation time. Previous studies have shown that a short preparation time leads not only to a generally impaired performance, but also to increased switch costs in error rates and RTs (Rogers and Monsell, 1995; Meiran, 1996). It was hypothesized that, if the efficiency of endogenous task-set control influences the susceptibility to task confusions, the proportion of distractor errors should be increased when preparation time is short (Meiran and Daichman, 2005).

### Materials and Methods

Twenty new participants (17 female) between 18 and 25 years of age (mean 21.1) with normal or corrected-to-normal vision participated in the study. Design and procedure of the experiment was the same as in Experiment 1 with the following exceptions. The cue was shown for 200 ms only. The cue-stimulus interval (CSI) was either 300 ms (short CSI condition) or 1000 ms (long CSI condition), and varied randomly within each block. The stimulus parts appeared synchronously and remained on the screen for 150 ms. The interval between the last response and the new cue was 1000 ms in the long CSI condition and 1700 ms in the short CSI condition.

### Results

#### RTs and Error Rates

Response times of correct responses and error rates were entered into a two-way ANOVA with repeated measurement on the variables Task Transition (task switch, task repetition), and CSI (long, short). The results are shown in Figure 1, right panel. For the RTs, significant main effects of Task Transition, *F*(1,19) = 65.6, *p* < 0.001, and CSI, *F*(1,19) = 66.1, *p* < 0.001, as well as a significant interaction between both variables, *F*(1,19) = 7.06, *p* < 0.05, was obtained. The switch costs were larger with a short CSI (118 ms) than with a long CSI (72 ms). For the error rates, significant main effects of Task Transition, *F*(1,19) = 67.5, *p* < 0.001, and CSI, *F*(1,19) = 12.8, *p* < 0.01, as well as a significant interaction between both variables, *F*(1,19) = 8.66, *p* < 0.01, was obtained, indicating that the switch costs were larger with a short CSI (7.2%) than with a long CSI (3.7%).

#### Proportion Distractor Errors

The mean proportion of distractor errors was 60.4%. This was significantly above 50%, *t*(19) = 6.26, *p* < 0.001, but not significantly different from the mean of Experiment 1, *F*(1,40) = 0.03, *p* = 0.87. A similar ANOVA revealed only a significant main effect of Task Transition (see, Figure 2, right panel), *F*(1,19) = 6.35, *p* < 0.05. The proportion of distractor errors was increased for task switches (62.7%) as compared to task repetitions (58.1%). However, neither a significant effect of CSI nor a significant interaction was obtained (*F*s<1).

#### Error Signaling

Again, the relative frequency of signaled errors (among all errors) and the latency of the signaling response was compared between non-distractor errors and distractor errors. Distractor errors (63.4%) were signaled less often than non-distractor errors (71.0%), *F*(1,19) = 11.5, *p* < 0.01. Signaling latencies were longer for distractor errors (652 ms) than for non-distractor errors (583 ms), *F*(1,19) = 8.75, *p* < 0.01.

### Discussion

Manipulating preparation time influenced performance in the expected direction. Decreasing preparation time led to increased RTs and error rates as well as to increased switch costs. However, the relative proportion of distractor errors was not affected by a reduced preparation time. This indicates that the additional errors due to a shorter preparation time were mainly response confusions. If these errors were task confusions, this would have led to an increased proportion of distractor errors among all errors. Because only response confusions were induced by a shorter preparation time, the same number of non-distractor errors and distractor errors was added which did not change the proportion of distractor errors. Accordingly, manipulating the efficiency of endogenous control seems to have no effect at all on the susceptibility to task confusions. Finally, the analysis of error signaling performance supported the results from the first experiment. Again, non-distractor errors were detected more frequently and more quickly than distractor errors.

A potential problem for the interpretation of our results is that our conditions differ with respect to target duration. While the target is presented for 150 ms in Experiment 2, it is presented for 250 ms in the target-first condition of Experiment 1 but for 150 ms in the distractor-first condition of Experiment 1. The question emerges whether these differences in target duration alone can account for differences in the proportion of distractor errors across conditions. In this case, one would expect that the mean proportion of distractor errors from Experiment 2 differs only from the target-first condition from Experiment 1, but not from the distractor-first condition from Experiment 1. However, our data speak against such an interpretation. The mean proportion from Experiment 2 (60.4%) lies almost exactly between that of the two conditions from Experiment 1 (target first: 56.5%, distractor first: 63.7%). Neither the difference between Experiment 2 and the target-first condition, *F*(1,40) = 2.09, *p* = 0.16, nor the difference between Experiment 2 and the distractor-first condition reached significance, *F*(1,40) = 1.40, *p* = 0.24. This suggests that target duration alone cannot explain our results.

## General Discussion

The present study investigated whether task confusions in task switching are due to failures in endogenous or exogenous control, respectively. As an indicator of the frequency of task confusions, the proportion of distractor errors relative to all errors in a three-choice paradigm was used. In Experiment 1, the efficiency of exogenous control was manipulated by varying the temporal order of target and distractor. When the distractor was presented prior to the target, the error rate was increased and this increase was mainly due to an increased number of distractor errors. Accordingly, reducing the efficiency of exogenous control increased the susceptibility of task confusions. In Experiment 2, the efficiency of endogenous control was manipulated by varying the time available for preparing the task in advance. When only 100 ms of preparation time was available, the error rate was strongly increased on task-switch trials. However, the proportion of distractor errors remained the same in this condition. Accordingly, reducing the efficiency of endogenous control did not increase the susceptibility to task confusions.

The finding that only exogenous control affected the frequency of task confusions has a number of implications regarding the nature of errors resulting from task confusions as well as the control processes that could prevent them: First of all, task confusions emerge because the stimulus activates the irrelevant task (see also Yamaguchi and Proctor, 2011), and therefore, are related to stimulus-induced task conflicts that were shown to prolong RTs on bivalent stimuli relative to univalent stimuli (Rogers and Monsell, 1995; Waszak et al., 2003; Steinhauser and Hübner, 2007, 2009). Because of this, errors due to task confusions correspond to, what has been called, *capture errors* (e.g., Norman, 1981), which are errors resulting when behavior is controlled by stimuli instead of internal goals. The pathologically increased frequency of capture errors due to frontal brain lesions is called *utilization behavior* (Lhermitte, 1983), and the present results suggest that task confusions could be a viable measure of this pathological behavior.

The finding that preparation time has no effect on the frequency of task confusions seems to contradict the results of Meiran and Daichman (2005). However, they observed such an effect under conditions where each task was associated with one hand, that is, when task confusions were considered to be the same as hand confusions. Therefore, their results could reflect the fact that motor preparation of the relevant hand is susceptible to failure when preparation time is short. Accordingly, these errors could have emerged because the correct task was performed with the wrong hand. In their second experiment, Meiran and Daichman (2005) came to a similar conclusion by applying multinomial modeling to a paradigm with overlapping response sets. The best fit was provided by a model that implied that task confusions but not response confusions are affected by preparation time. However, the results of multinomial modeling strongly depend on which models are compared. For instance, no model was tested that corresponded to the results of the present study. Such a model would assume that preparation time affected the rate of response confusions but not the rate of task confusions. Thus, it is unclear whether the present results contradict the empirical data of Meiran and Daichman (2005), or whether they only contradict their model.

The present findings also seem to contradict theories that attribute task confusions to a failure in establishing the correct task set. For instance, the model of Altmann and Gray (2008) assumes task confusions to be increased on task-switch trials when preparation time is short. The present results rather suggest that additional errors under these conditions are due to an increased susceptibility to response confusions. This could imply that error switch costs reflect a similar mechanism as RT switch costs, that is, a more difficult response selection due to more interference or a less prepared task set. However, our data mainly showed that the susceptibility to task confusions is independent from the efficiency of endogenous preparation (Meiran et al., 2002). Whereas it is still possible that task confusions are related to a failed preparation processes, they seem to be largely unaffected by preparation time. This conclusion receives further support from recent studies showing that also the task congruency effect, an index of conflict between tasks, is independent of preparation time (e.g., Yamaguchi and Proctor, 2011; Schneider, 2015).

Further implications of the present results concern the control processes that can prevent task confusions. If task confusions emerge because an irrelevant stimulus element activates a task, selective attention should be a viable means to prevent task confusions. This suggests that task confusions should be less frequent under conditions that allow for a more efficient visual selective attention. Indeed, Steinhauser and Hübner (2009) found that stimulus-induced task conflicts are reduced when spatial selective attention can be applied to select the relevant stimulus feature. This also supports the idea that a main function of task-set reconfiguration consists in configuring the attentional set (Meiran, 2000; Meiran et al., 2008).

Finally, our results also indicate that errors due to task confusions are less detectable than errors due to response confusions. This makes sense in the light of current theories of error detection. For instance, Yeung et al. (2004) proposed that error detection is based on the detection of a post-error response conflict that emerges between the still activated error response and the later activated correct response. Similarly, Steinhauser et al. (2008) suggested that error detection is based on the detection of an internal error correction which occurs when the correct response exceeds the response threshold after the error response (see also, Rabbitt and Vyas, 1981). Despite the differences, both theories assume that the efficiency of error detection depends on how strongly the correct response becomes activated after an error. This could explain why errors due to task confusions are more difficult to detect. The correct response can only become activated after an error when the correct task set is activated. When an error occurs because of a task confusion, then the wrong task set is activated at the time of the error. Detection of this error requires that the system eventually succeeds in activating the correct task set. Since this should fail with a certain probability, error detection is less likely for errors due to task confusions than for errors due to response confusion.

Taken together, the results of the present study suggest that failures of exogenous control are the primary source of task confusions emerging when participants rapidly switch between tasks that share the same stimuli and responses. Under these conditions, task confusions occur when the correct task set is prepared but the wrong task set is activated by the stimulus. In contrast, the efficiency of endogenous control seems to play a minor role only.

## Author Contributions

MS designed and performed research; MS analyzed data; MS and MG interpreted the data and wrote the paper.

### Conflict of Interest Statement

The authors declare that the research was conducted in the absence of any commercial or financial relationships that could be construed as a potential conflict of interest.
